# Editorial: Translational approaches to combat emerging viral infections: diagnosis, immunopathogenesis, and therapeutics

**DOI:** 10.3389/fcimb.2024.1406240

**Published:** 2024-04-05

**Authors:** Gopinathan Pillai Sreekanth, Gathsaurie Neelika Malavige

**Affiliations:** ^1^ Department of Applied Biology, CSIR-Indian Institute of Chemical Technology, Tarnaka, Hyderabad, Telangana, India; ^2^ Allergy Immunology and Cell Biology Unit, Department of Immunology and Molecular Medicine, University of Sri Jayewardenepura, Nugegoda, Sri Lanka; ^3^ MRC Human Immunology Unit, MRC Weatherall Institute of Molecular Medicine, University of Oxford, Oxford, United Kingdom

**Keywords:** viral infection, translational research, diagnosis, immunopathogenesis, therapeutics

Several novel viral infections have caused global outbreaks in the past. However, the recent severe acute respiratory syndrome coronavirus-2 (SARS-CoV-2) developed as a pandemic, disrupting global human health and affected the economy. The SARS-CoV-2 has been reported to show continuous changes in the genetic code, due to rapid mutations, giving rise to many variants of concern (VOCs). Due to mutations within primer binding site, diagnosing these variants from the clinical samples of SARS-CoV-2 infected patients has also been challenging. Understanding the immunopathogenesis of the emerging SARS-CoV-2 variants is crucial development of preventive or therapeutic strategies. Many novel vaccine candidates were established to combat SARS-CoV-2 infection. However, most of them subsequently demonstrated reduced efficacy to in preventing infection due to the emerging SARS-CoV-2 VOCs, although they have remained effective against severe disease and hospitalizations. A large number of compounds were shown to interfere with the SARS-CoV-2 life cycle or initiate host responses to thereby reducing viral load in the preclinical studies. However, as many such compounds failed to show efficacy in clinical trials, it would be important to carefully validate all novel and repurposed compounds in randomized controlled trials. Evaluating the efficacy of these candidate drugs in the contect of natural infection of SARS-CoV-2 is currently impossible, and not required as the global population have either been naturally infected, vaccinated and in most instances, both. Several neutralizing antibodies to SARS-CoV-2 have been identified, and most of them target the receptor binding domain (RBD), N-terminal domain (NTD), and S2 domain of the SARS-CoV-2 spike protein. Hence, translational approaches targeting those critical gaps might pave the way for the rapid diagnosis and development of therapeutic strategies to combat emerging SARS-CoV- variants.

The US Centers for Disease Control (CDC) and World Health Organization (WHO) release opportune warnings through notices/bulletins on emerging and reemerging infections worldwide. However, the diagnosis, immunopathogenesis, and designing therapeutic strategies for these viral infections were challenging. The themes covered in this Research Topic focused on novel translational research aspects in the diagnosis, immunopathogenesis, and therapeutic designs to combat recent emerging viral diseases. The nomogram model was widely used to represent disease prediction, which explicitly determines the association of a specific disease with its risk factors through a graphical scoring tool. Chen et al. compared the calibration curves obtained through the SARS-CoV-2 infection prediction tool with the actual observations from a SARS-CoV-2 retrospective study. The data from the 6189 vaccinated individuals in the Fujian province of China during the delta variant outbreak in September 2021 were analyzed. The authors developed and validated the nomogram data using univariate and multivariate regression analysis, representing the SARS-CoV-2 breakthrough probability using a calibration plot; however, this study was conducted with a small sample size for the number of SARS-CoV-2 breakthrough cases. This method may be further evaluated in a higher sample size, which would benefit the management of SARS-CoV-2 breakthrough infection. In another study by Zhang et al., a lung mask-weighted global average pooling-based (GAP-based) deep learning model was developed to differentiate normal and pneumonia cases with the SARS-CoV-2 infection. This machine-learning algorithm for the chest computed tomography (CT) images could diagnose SARS-CoV-2 infection triage with a sensitivity of 96.5% and specificity of 87.88%. This method would benefit a more sensible diagnosis of SARS-CoV-2 triage using chest CT scan results; however, this method is not an applicable quantitative method. The authors also pointed out that respiration and heart motion may affect the accuracy of this learning method.

Metagenomic Next Generation Sequencing (mNGS) was used to diagnose pathogens in patients with fevers of unknown origin (FUO). Song et al. correlated this method with diagnosing positive cases of Epstein-Barr virus (EBV) associated malignancies. In this preliminary study conducted on 29 EBV-positive participants, mNGS displayed 90% sensitivity and 89.5% specificity in identifying the EBV-associated tumors; however, a more detailed evaluation on a larger sample size is necessary before its clinical practice.

The severity of the SARS-CoV-2 pandemic and the higher death toll in developed nations have made a significant impact on the development of prevention and treatment strategies for SARS-CoV-2 infection. Several small molecules targeting the viral proteins (mostly interfering with the viral enzymes) or host proteins (mainly the cell receptors contributing to viral attachment and internalization) were evaluated in preclinical and clinical studies. Azvudine was the first double-target nucleoside drug developed as an anti-SARS CoV-2 agent by China, and the National Medical Products Administration (NMPA) has approved its emergency use to treat SARS-CoV-2 infection based on its efficacy in randomized controlled trials. Mao et al. identified the level of lactate dehydrogenase (LDH) as a predictive marker to evaluate the effectiveness of Azvudine in the treatment of SARS-CoV-2 infection. The authors identified in their retrospective study conducted in the SARS-CoV-2 infected clinical samples treated with or without Azvudine that the higher level of LDH was found to worsen the SARS-CoV-2 progression with other outcome measures. For the first time, this study identified LDH as a disease progression predictor in SARS-CoV-2 infected patients treated with Azvudine; however, more detailed studies with the isoforms of LDH warrant more insights, mainly identifying the source of higher LDH levels. Neutralizing antibodies are another promising treatment strategy that has been evaluated for its efficacy in combating SARS-CoV-2 infection. Bajpai et al. reviewed the recent progress in the novel neutralizing antibodies to combat human coronaviruses, including the recently emerged SARS-CoV-2. The authors concluded that targeting the conserved epitopes on the spike protein may be the best approach for identifying effective neutralizing antibodies against pan-SARS-CoV-2 VOCs.

Overall, this Research Topic discussed the recent translational developments in viral infections, mainly the diagnostic and therapeutic strategies for SARS-CoV-2 infection. More detailed studies on rapid kits and clinical markers may ease the diagnosis. Artificial intelligence-based prediction tools may illustrate the disease progression and severity. The lessons learned from the recent SARS-CoV-2 pandemic may aid in developing translational approaches to combat emerging and reemerging viral infections.

## Author contributions

GS: Writing – original draft, Writing – review & editing. GM: Writing – original draft, Writing – review & editing.

